# Gestational weight loss and fetal growth in uncomplicated pregnancies among women with obesity: a population-based retrospective cohort study

**DOI:** 10.1038/s41366-023-01382-6

**Published:** 2023-10-13

**Authors:** Yanfang Guo, Sara C. S. Souza, Liam Bruce, Rong Luo, Darine El-Chaâr, Laura M. Gaudet, Katherine Muldoon, Steven Hawken, Sandra I. Dunn, Alysha L. J. Dingwall-Harvey, Mark C. Walker, Shi Wu Wen, Daniel J. Corsi

**Affiliations:** 1 Clinical Epidemiology Program, Ottawa Hospital Research Institute, Ottawa, ON Canada; 2 BORN Ontario, Children’s Hospital of Eastern Ontario, Ottawa, ON Canada; 3 School of Epidemiology and Public Health, University of Ottawa, Ottawa, ON Canada; 4 Department of Obstetrics, Gynecology & Newborn Care, The Ottawa Hospital, Ottawa, ON Canada; 5 Department of Obstetrics and Gynecology, University of Ottawa, Ottawa, ON Canada; 6 Department of Obstetrics and Gynecology, Kingston Health Sciences Centre, Kingston, ON Canada; 7 Department of Obstetrics and Gynecology, Queen’s University, Kingston, ON Canada; 8 Children’s Hospital of Eastern Ontario Research Institute, Ottawa, ON Canada; 9 School of Nursing, University of Ottawa, Ottawa, ON Canada; 10 International and Global Health Office, University of Ottawa, Ottawa, ON Canada

**Keywords:** Weight management, Epidemiology

## Abstract

**Objective:**

The impact of gestational weight loss (GWL) on fetal growth among women with obesity remains unclear. This study aimed to examine the association between weight loss during pregnancy among women with body mass index (BMI) ≥ 30 kg/m^2^ and the risk of small-for-gestational-age (SGA) and large-for-gestational-age (LGA) neonates.

**Methods:**

We conducted a retrospective, population-based cohort study of women with pre-pregnancy obesity that resulted in a singleton live birth in 2012-2017, using birth registry data in Ontario, Canada. Women with pregnancy complications or health conditions which could cause weight loss were excluded. GWL is defined as negative gestational weight change (≤0 kg). The association between GWL and fetal growth was estimated using generalized estimating equation models and restricted cubic spline regression analysis. Stratified analysis was conducted by obesity class (I:30–34.9 kg/m^2^, II:35–39.9 kg/m^2^, and III + : ≥40 kg/m^2^).

**Results:**

Of the 52,153 eligible women who entered pregnancy with a BMI ≥ 30 kg/m^2^, 5.3% had GWL. Compared to adequate gestational weight gain, GWL was associated with an increased risk of SGA neonates (aRR:1.45, 95% CI: 1.30–1.60) and a decreased risk of LGA neonates (aRR: 0.81, 95% CI:0.73–0.93). Non-linear L-shaped associations were observed between gestational weight change and SGA neonates, with an increased risk of SGA observed with increased GWL. On the contrary, non-linear S-shaped associations were observed between gestational weight change and LGA neonates, with a decreased risk of LGA observed with increased GWL. Similar findings were observed from the stratified analysis by obesity class.

**Conclusion:**

These findings highlight that GWL in women with obesity may increase the risk of SGA neonates but reduce the risk of LGA neonates. Recommendations of GWL for women with obesity should be interpreted with caution.

## Introduction

Obesity, commonly defined as body mass index (BMI) greater than or equal to 30 kg/m^2^, is a significant health concern that affects over 14 million women worldwide entering pregnancy each year [1]. The risks of adverse outcomes related to living with obesity prior to or during pregnancy have been documented for both pregnant women and their offspring [2], including an increased risk of pregnancy-induced hypertension, gestational diabetes mellitus [3], and poorer offspring cardiovascular health throughout the life course [4].

Appropriate weight management during pregnancy has been suggested as one of the few modifiable factors with the potential to minimize adverse perinatal outcomes [5]. The 2009 Institute of Medicine (IOM) guidelines for gestational weight gain recommend that women with pre-pregnancy obesity should gain between 5 and 9 kg during pregnancy, while a weight gain between 11.5 and 16 kg is indicated for women with normal weight BMI [6]. However, there is no tailoring of gestational weight gain recommendations by the severity of obesity and no clear guidelines for gestational weight loss (GWL) for women living with extreme obesity. Although weight loss during pregnancy is not recommended by the IOM guidelines, studies have reported that 5–8% of women with obesity in general [7, 8] and up to 15% of women with extreme obesity experienced intentional GWL [9, 10].

The association between GWL and fetal outcomes among women with high BMI, including small-for-gestational-age (SGA) and large-for-gestational-age (LGA) neonates, remains controversial. In a systematic review conducted in 2014, it was reported that women with obesity who experienced GWL had higher odds of SGA (<10th percentile) but lower odds of LGA (>90th percentile) neonates when compared to gestational weight gain within the guidelines [11]. However, the cohort studies included in the systematic review have significant limitations, such as small sample size, absence of important confounders, lack of uniformity in defining obesity classes, and scarce information on maternal health problems that could cause unintended weight loss (e.g., maternal pre-existing health conditions, pregnancy complications, psychosocial factors such as anxiety and depression) [9, 12–16]. Additionally, emerging evidence suggested that the current recommendation of a weight gain of 5–9 kg is still too high for women with extreme obesity, and very limited weight gain, no weight gain, or even weight loss during pregnancy has been found to be safe and effective through regular quality checks and professional training sessions [17].

There are numerous studies that deal with gestational weight change data as a categorical variable. However, the categorization of a numeric variable, such as gestational weight gain, leads to multiple issues, including loss of information, discontinuity in the estimated average outcome value when moving from one category to another, and difficulties with comparing results across studies. To precisely estimate the association between GWL and fetal growth, this study used regression analysis with restricted cubic splines. Our aim was to use Ontario’s world-leading birth registry data to investigate the impact of GWL on fetal growth among women with uncomplicated pregnancies who have obesity and to fill the current knowledge gap, providing guidance on weight management during pregnancy.

## Materials and methods

### Study design and data sources

This population-based retrospective cohort study was reviewed and approved by the Children’s Hospital of Eastern Ontario (19/09PE) and Ottawa Health Science Network (20190704-01 K) Research Ethics Boards. The study cohort was assembled from Better Outcomes Registry & Network (BORN) data in Ontario, Canada (https://www.bornontario.ca/en/about-born/), from April 1, 2012, to March 31, 2017. As the largest and most robust provincial perinatal dataset in Canada, BORN Ontario captures all hospital births in the province, accounting for 40% of births across Canada [18, 19]. Based on the BORN 2016 report, roughly 18% of women start pregnancy with obesity, totaling around 126,000 individuals for our 5-year cohort. Within this cohort, using preliminary BORN Ontario 2012–2017 data, we expected at least 2200 women with obesity to fall into the GWL group with low-risk conditions. With a sample size of 2200 and considering at least 5% prevalence of SGA and LGA neonates, we can achieve over 95% statistical power to detect a 30% or greater increase in the risk of SGA and LGA neonates in the GWL group compared to those with optimal weight gain, all with a two-tailed alpha of 5%.

Maternal demographic and clinical information related to pregnancy, labour and birth, and postpartum complications have been routinely collected through medical records, clinical forms, and patient interviews when pregnant women are admitted to the hospital for delivery. The data quality has been assured by regular quality checks and professional training sessions, and a high level of accuracy and completeness have been demonstrated through previous validation studies [19, 20]. In addition, BORN records were externally linked to the Canadian Institute for Health Information’s (CIHI) Discharge Abstract Database (DAD) to improve ascertainment of some maternal health conditions which cause significant weight loss during pregnancy (conditions were listed in the section of study population). As a national healthcare administrative database, CIHI-DAD collects each abstract from hospital discharge, which contains demographics, medical diagnosis and medical interventions. Medical diagnoses are coded by the Canadian implementation of the International Classification of Diseases, 10th Revision (ICD-10-CA) and interventions are coded by the Canadian Classification of Health Interventions (CCI). Neighbourhood level income and education data from 2011 Canada Census were linked to BORN records using the Postal Code Conversion File (PCCF).

### Study population

All pregnant women with a pre-pregnancy BMI of 30 kg/m^2^ or higher who gave birth to a singleton live infant with a birthweight of more than 500 grams or a gestational age of over 22 weeks were initially included in this study. However, women with any of the following conditions were excluded: gestational age at delivery >42 weeks, maternal age <19 years old, and multiple pregnancies. Moreover, pregnant women with health conditions that could cause unintended weight loss were removed from the cohort, including tuberculosis, hepatitis, HIV, cancer, hypertension, autoimmune disorder, diabetes, hypothyroidism, hyperthyroidism or other endocrine disorder, anxiety, depression, eating disorders, and gastrointestinal diseases (BORN and ICD-10 codes are provided in Supplementary Table 1). Additionally, pregnancies with any fetal congenital anomalies that may affect birth weight were removed from the cohort, such as suspected lethal anomaly (e.g., skeletal dysplasia including achondrogenesis) or non-lethal anomaly (e.g., omphalocele), genetic syndrome (e.g., Beckwith Wiedemann Syndrome), or chromosomal anomaly (e.g., Down Syndrome, Trisomy 18). Cases with other pregnancy complications (i.e., gestational diabetes, pregnancy-induced hypertension, and infections during pregnancy) were also excluded. Observations with missing data on BMI or implausible weight data were removed to further restrict our analysis.

### Measures

#### Outcome variable

The outcomes of interest were SGA and LGA neonates. In the primary analysis, SGA was defined as birth weight <10th percentile for the same gestational age, while LGA was defined as birth weight >90th percentile for the same gestational age, according to the sex-specific Canadian birth weight reference for singletons [21]. Supplementary analyses were performed to assess the association between GWL and extreme neonatal outcomes, specifically SGA <3rd percentile and LGA >97th percentile.

#### Exposure variables

Total weight change during pregnancy was the exposure variable of interest. Gestational weight change was derived from the difference between maternal weight at delivery and pre-pregnancy weight, as recorded in the registry. GWL is defined as negative gestational weight change (≤0 kg).

Based on IOM guidelines for the population with obesity, women who gained 5–9 kg and delivered at term (≥37 gestational weeks) were classified as gaining adequate gestational weight. Women who had gestational weight gain lower (no weight loss) or higher than the recommended weight gain were classified as having inadequate or excessive weight gain, respectively. For women with preterm birth (<37 gestational weeks), the duration of gestation was accounted for in our calculations. The range for adequate weight gain in women with preterm birth was calculated based on IOM recommendations for the amount of weight gain during the first trimester (0.5 kg for individuals with obesity) plus the amount of weight gain during the second and third trimester [between (gestational age - 13) × (0.17 kg/week) and (gestational age - 13) × (0.27 kg/week)] [22].

GWL was treated as both continuous and categorical exposure variables in the analysis. When coding the gestational weight change as a categorical variable, weight change throughout pregnancy was classified into four categories: weight loss, inadequate weight gain, adequate weight gain, and excessive weight gain. The reference category was adequate weight gain group.

#### Covariables and potential confounders

Several covariates and potential confounders were selected based on the literature, review of a directed acyclic graph (DAG), and data availability: pre-pregnancy BMI, maternal age at delivery, gestational age, parity, neighbourhood education quintile, neighbourhood household median income quintile, conception type, smoking, and antenatal health care provider.

#### Effect modifier

Pre-pregnancy obesity class was considered as the effect modifier for the association between GWL and fetal growth. Pre-pregnancy obesity groups were identified using pre-pregnancy BMI, calculated by self-reported pre-pregnancy weight and self-reported height, and were classified according to the World Health Organization standards as follows: Class I obesity (BMI 30.0–34.9 kg/m^2^), Class II obesity (BMI 35.0–39.9 kg/m^2^), Class III obesity (BMI 40.0–49.9 kg/m^2^), Class IV obesity (BMI 50.0–59.9 kg/m^2^), and Class V obesity (BMI ≥ 60 kg/m^2^).

### Statistical analysis

#### Descriptive analysis

Summary statistics were generated to describe the pregnant women with obesity for four groups based on gestational weight change: GWL, inadequate weight gain, adequate weight gain, and excessive weight gain. The variables included in the analysis were social-demographic, behaviour, medical/obstetric, and delivery factors. Continuous variables were described by mean ± standard deviation (SD). Categorical variables were described by count and percent (%). Prevalence of overall GWL was examined for women with obesity, and by obesity class (I, II, and III+).

#### Association between GWL and risk of SGA and LGA neonates

Univariate regression analyses were conducted to assess the association between potential confounders and occurrence of SGA and LGA neonates. Multivariable modified Poisson [23] regression models were further developed to estimate adjusted risk ratios (aRR) and 95% confidence intervals (CIs) for the association between gestational weight change and risk of SGA and LGA neonates, with the adequate weight gain group as reference. The multivariable models adjusted for maternal age, gestational age, pre-pregnancy BMI, parity, neighbourhood household median income quintile, smoking, and antenatal health care provider, and accounted for repeated pregnancies during the 2012–2017 period using generalized estimation equation (GEE) methods to adjust for variance. Multiple imputations methods were used to account for the missing data of covariates and confounders in the regression models. Five complete datasets were imputed by using the fully conditional specification (FCS) methods. Maternal age, gestational age, and pre-pregnancy BMI were imputed using a linear regression model, while parity (nulliparous, multiparous), neighbourhood-level income (quintile 1–5), smoking during pregnancy (yes, no), and antenatal healthcare provider (inclusive of obstetrician [yes/no]) were imputed using logistic regression models. The results of the analyses from the five imputed datasets were combined in accordance with Rubin’s rules to account for uncertainty due to imputation.

Stratified analyses were implemented to explore the association between GWL and SGA and LGA neonates by obesity class and interaction effects between pre-pregnancy BMI groups and GWL on SGA and LGA neonates were also assessed using Wald tests at *p* ≤ 0.05 level.

#### Non-linear dose-response relationship between weight change and risk of SGA and LGA neonates

The non-linear associations of gestational weight change and risk of SGA and LGA neonates were examined by using modified Poisson regression models with restricted cubic spline terms to represent weight change in pregnancy. This analysis was conducted using the first imputed dataset. Absolute gestational weight change and weekly average gestational weight change were modelled using restricted cubic splines, with 5 knots placed at quantiles recommended by Harrell: 0.05, 0.275, 0.50, 0.725, 0.950 [24]. These regression models were adjusted for the confounders specified in the categorical analysis. RRs for SGA and LGA neonates were calculated as the predicted probability of the outcome (SGA or LGA neonates) in the regression model divided by the predicted probability at a chosen reference value, which was set to the total kilogram change (i.e., 7.0 kg) or weekly average weight change (i.e., 0.22 kg). 95% CIs for the RRs were calculated using the bootstrap percentile method with 1000 replicates [25].

#### Sensitivity analysis

A sensitivity analysis was conducted to assess the impact of our analytical methods on the findings. Firstly, we performed a complete case analysis to assess the effectiveness of the multiple imputation approach. Secondly, we examined the associations between GWL and extreme SGA (<3rd percentile) and LGA (>97th percentile) neonates to test the robustness of our main results.

## Results

A total of 52,153 women met our inclusion criteria and were included in the analysis. Differences in sociodemographic, behavioural, medical/obstetrical, and delivery factors among women in GWL, inadequate weight gain, adequate weight gain, and excessive weight gain are described in Table 1.

**Table 1:** Demographic characteristics of women with obesity and uncomplicated pregnancies resulting in birth in Ontario between 2012-2017, by gestational weight change (*n* = 52,153).

Characteristic	GWL		Inadequate weight gain		Adequate weight gain		Excessive weight gain		Total (*N* = 52,153)		*P*
	*N*	% (col)	*N*	% (col)	*N*	% (col)	*N*	% (col)	*N*	% (col)	
Pre-pregnancy BMI (kg/m2) (Mean ± SD)	(37.4 ± 6.2)		(36.1 ± 5.1)		(35.2 ± 4.6)		(30.5 ± 4.8)		(34.9 ± 4.9)		<0.0001
Class I obesity (30.0–34.9)	1171	42.4	3029	50.4	3586	58.3	25,053	67.3	32,839	63.0	
Class II obesity (35.0–39.9)	819	29.7	1767	29.4	1704	27.7	8239	22.1	12,529	24.0	
Class III obesity (40.0–44.9)	445	16.1	844	14	603	9.8	2577	6.9	4469	8.6	
Class IV obesity (45.0–49.9)	218	7.9	258	4.3	184	3	789	2.1	1449	2.8	
Class V obesity (≥50.0)	108	3.9	114	1.9	70	1.1	575	1.5	867	1.7	
Maternal age at delivery (years) (Mean ± SD)	(29.9 ± 5.1)		(30.3 ± 5.1)		(30.7 ± 5.1)		(30.5 ± 4.9)		(30.4 ± 5.0)		<0.0001
19–34	2259	81.8	4697	78.1	4729	76.9	29,342	78.8	41,027	78.7	
35–39	400	14.5	1080	18	1143	18.6	6540	17.6	9163	17.6	
≥40	102	3.7	235	3.9	275	4.5	1351	3.6	1963	3.8	
Neighbourhood education quintile (percentage of university degrees among adults 25–64 years old)											<0.0001
quintile 1 (lowest)	666	25.0	1547	26.8	1444	24.5	8202	22.9	11,859	23.7	
quintile 2	676	25.4	1357	23.5	1422	24.1	8586	24.0	12,041	24.0	
quintile 3	666	25.0	1299	22.5	1333	22.6	8196	22.9	11,494	22.9	
quintile 4	447	16.8	1074	18.6	1151	19.5	7204	20.1	9876	19.7	
quintile 5 (highest)	207	7.8	487	8.4	543	9.2	3610	10.1	4847	9.7	
Missing	99	3.6	248	4.1	254	4.1	1435	3.9	2036	3.9	
Neighborhood household median income quintile											<0.0001
quintile 1 (lowest)	826	31.3	1664	29.1	1619	27.7	8783	24.8	12,892	26.0	
quintile 2	535	20.3	1120	19.6	1220	20.9	6893	19.4	9768	19.7	
quintile 3	522	19.8	1175	20.6	1141	19.5	7198	20.3	10,036	20.2	
quintile 4	496	18.8	1155	20.2	1190	20.4	8021	22.6	10,862	21.9	
quintile 5 (highest)	262	9.9	600	10.5	668	11.4	4570	12.9	6100	12.3	
Missing	120	4.3	298	5.0	309	5.0	1768	4.7	2495	4.8	
Parity											<0.0001
Nulliparous	742	27.0	1545	25.8	1662	27.1	13,909	37.5	17,858	34.4	
Multiparous	2007	73.0	4449	74.2	4464	72.9	23,186	62.5	34,106	65.6	
Missing	12	0.4	18	0.3	21	0.3	138	0.4	189	0.4	
Conception type											0.155
In vitro fertilization	26	1.0	51	0.9	61	1.0	440	1.2	578	1.1	
Intrauterine insemination	45	1.7	101	1.7	105	1.7	696	1.9	947	1.9	
No assisted reproductive technology	2617	97.4	5715	97.4	5843	97.2	35,168	96.9	49,343	97.0	
Missing	73	2.6	145	2.4	138	2.2	929	2.5	1285	2.5	
Smoking during pregnancy											<0.0001
No	2256	83.0	5053	85.5	5353	88.6	32,576	89.2	45,238	88.4	
Yes	461	17.0	854	14.5	690	11.4	3928	10.8	5933	11.6	
Missing	44	1.6	105	1.7	104	1.7	729	2.0	982	1.9	
Maternal pre-existing health conditions (chronic hypertension, diabetes, chronic heart disease, pulmonary disease)											0.2076
Yes	2570	93.1	5649	94	5791	94.2	35,002	94	49,012	94	
No	191	6.9	363	6	356	5.8	2231	6	3141	6	
Antenatal health care provider											0.0041
Inclusive of obstetrician	2109	77.9	4582	77.6	4560	75.4	27,758	76.0	39,009	76.2	
Does not include obstetrician	600	22.1	1322	22.4	1488	24.6	8753	24.0	12,163	23.8	
Missing	52	1.9	108	1.8	99	1.6	722	1.9	981	1.9	
Gestational age at delivery (weeks) (Mean ± SD)	(39.1 ± 2.5)		(39.3 ± 1.9)		(39.5 ± 1.6)		(39.5 ± 1.7)		(39.4 ± 1.8)		<0.0001
<37	228	8.3	348	5.8	270	4.4	1781	4.8	2627	5	
≥37	2533	91.7	5664	94.2	5877	95.6	35,452	95.2	49,526	95	

Data source: BORN-CIHI linked data 2012-2017. *SD* standard deviation; *BMI* body mass index; *GWL* gestational weight loss.

*SD* standard deviation, *BMI* body mass index, *GWL* gestational weight loss.

Data were extracted from the BORN Information System (BIS) on April 1, 2021.

Missing data were excluded from the percentage calculations (i.e. N of numerator/[N of denominator - N of missing]).

**Fig. 1:**
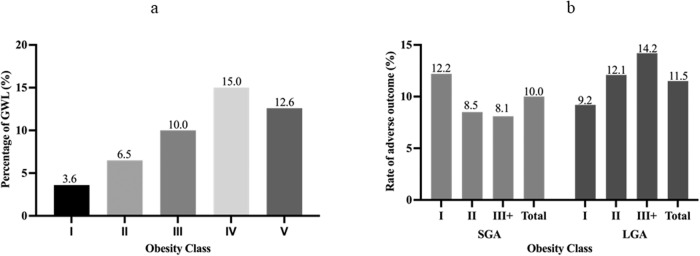
Prevalence of GWL, SGA and LGA neonates by obesity class. **a** Prevalence of GWL by obesity class, Ontario, 2012–2017 (*n* = 52,153). **b** Prevalence of SGA (<10th percentile) and LGA (>90th percentile) neonates among women with GWL by obesity class, Ontario, 2012–2017 (*n* = 2761).

Among all eligible women, 2761 (5.3%) experienced GWL during their pregnancy. The majority of women (63.0%) were classified as class I obesity, followed by 24.0%, 8.6%, 2.8% and 1.7% for class II, III, IV and V obesity. The rate of GWL was 3.6%, 6.5%, 10.0%, 15.0% and 12.6% for obesity class I, II, III, IV and V, respectively (Fig. 1a). Overall, the rates of SGA (<10th percentile) and LGA (>90th percentile) neonates among women with GWL were 10.0% and 11.5%. The rates of SGA (<10th percentile) neonates by obesity class were 12.2%, 8.5%, and 8.1% for classes I, II, and III+, respectively. The rates of LGA (>90th percentile) neonates by obesity class were 9.2%, 12.1%, and 14.2% for classes I, II, and III+, respectively (Fig. 1b).

**Fig. 2:**
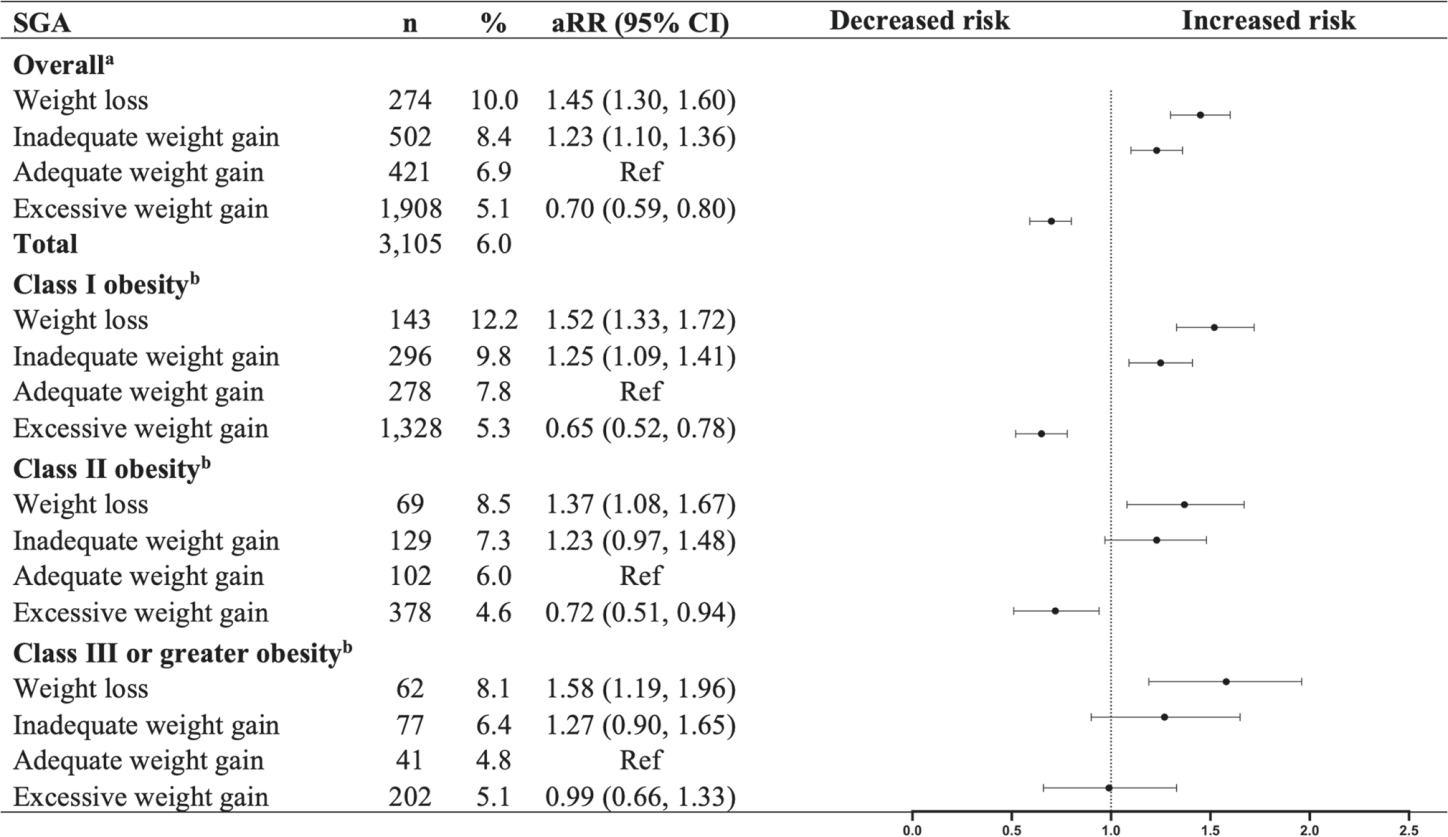
Association between weight change during pregnancy and risk of SGA (<10th percentile) neonates. SGA small for gestational age, aRR adjusted relative risk, CI confidence interval. ^a^Models were adjusted for maternal age, gestational age, pre-pregnancy BMI, parity, neighbourhood-level income, smoking, and antenatal health care provider. ^b^Models were adjusted for maternal age, gestational age, parity, neighbourhood-level income, smoking, and antenatal health care provider.

**Fig. 3:**
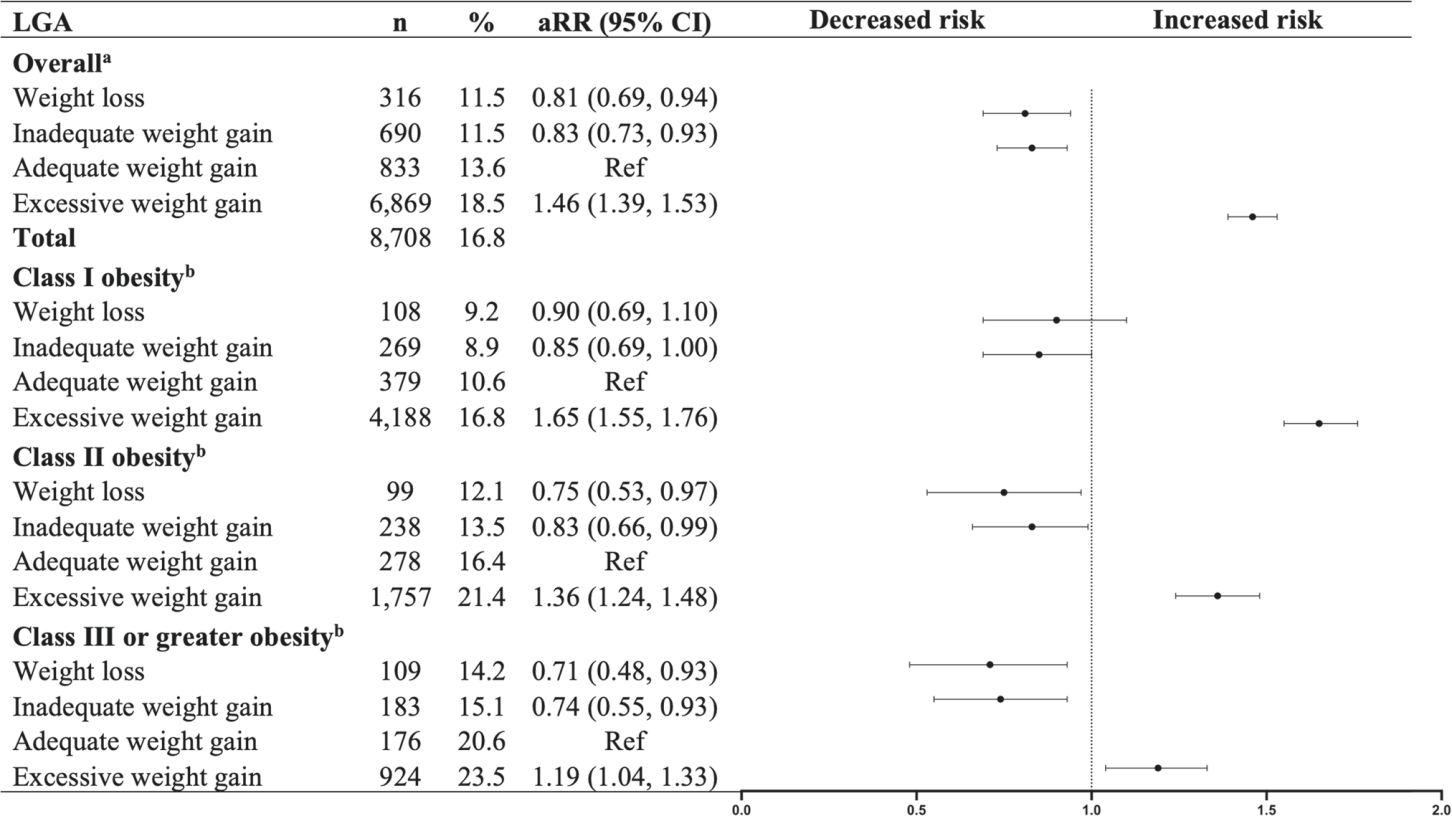
Association between weight change during pregnancy and risk of LGA (>90th percentile) neonates. LGA large for gestational age, aRR adjusted relative risk, CI confidence interval. ^a^Models were adjusted for maternal age, gestational age, pre-pregnancy BMI, parity, neighbourhood-level income, smoking, and antenatal health care provider. ^b^Models were adjusted for maternal age, gestational age, parity, neighbourhood-level income, smoking, and antenatal health care provider.

After adjusting for confounders and imputing missing data, GWL in women with obesity was associated with an increased risk of neonatal SGA (<10th percentile) (aRR: 1.45, 95% CI: 1.30–1.60) and a decreased risk of neonatal LGA (>90th percentile) (aRR: 0.81, 95% CI: 0.69–0.94), compared to adequate gestational weight gain (see Figs. 2 and 3). The interaction term for obesity class and weight gain categories was significant (Wald *p* < 0.01). In women who entered pregnancy with obesity, GWL was significantly associated with an increased risk of SGA (<10th percentile) neonates, regardless of obesity class (I: aRR: 1.52, 95% CI: 1.33–1.72; II: aRR: 1.37, 95% CI: 1.08–1.67; III+: aRR: 1.58, 95% CI: 1.19–1.96). In contrast, GWL was associated with a decreased risk of LGA (>90th percentile) neonates by 25% (aRR: 0.75, 95% CI 0.53–0.97) and 29% (aRR: 0.71, 95% CI 0.48–0.93) in women with obesity classes II and III+, respectively.

The sensitivity analysis using complete case datasets showed similar results (refer to Supplementary Table 2). Further analysis assessing extreme outcomes revealed that GWL was linked with an increased risk of SGA <3rd percentile (aRR: 1.59, 95% CI 1.30–1.88), but the decreased risk of LGA >97th percentile (aRR: 0.82, 95% CI 0.59–1.06) was not statistically significant. The findings for the association between GWL and extreme outcomes are in line with our primary results for SGA; although there was a non-significant trend observed for LGA. The associations by obesity class were not assessed due to insufficient sample size for each stratum (see Supplementary Tables 3 and 4).

**Fig. 4:**
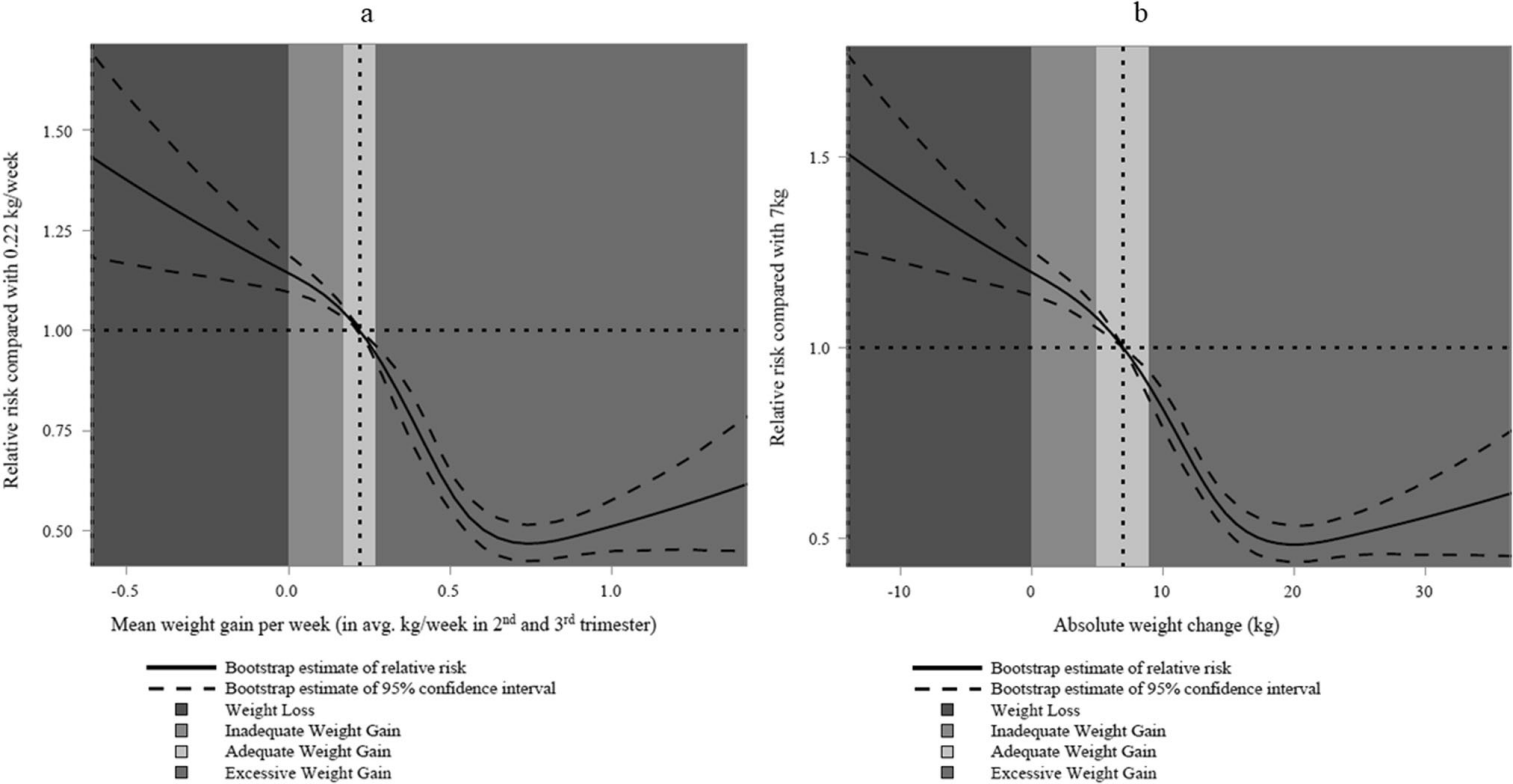
Dose-response relationship between weight change and risk of SGA (<10th percentile) neonates. **a**, **b** Dose-response relationship between weight change during pregnancy and risk of SGA (<10th percentile) neonates. RRs and 95% CIs of SGA (<10th percentile) were calculated with respect to total gestational weight change in kilograms (**a**) and average gestational weight change per week (**b**). Results from multivariable regression models using generalized estimation equation (GEE) methods and restricted cubic splines with 5 knots. Model adjusted for maternal age, gestational age, pre-pregnancy BMI, parity, neighbourhood household median income quintile, smoking, and antenatal health care provider. Each plot was centred to display 99% of gestational weight change values in the graph.

**Fig. 5:**
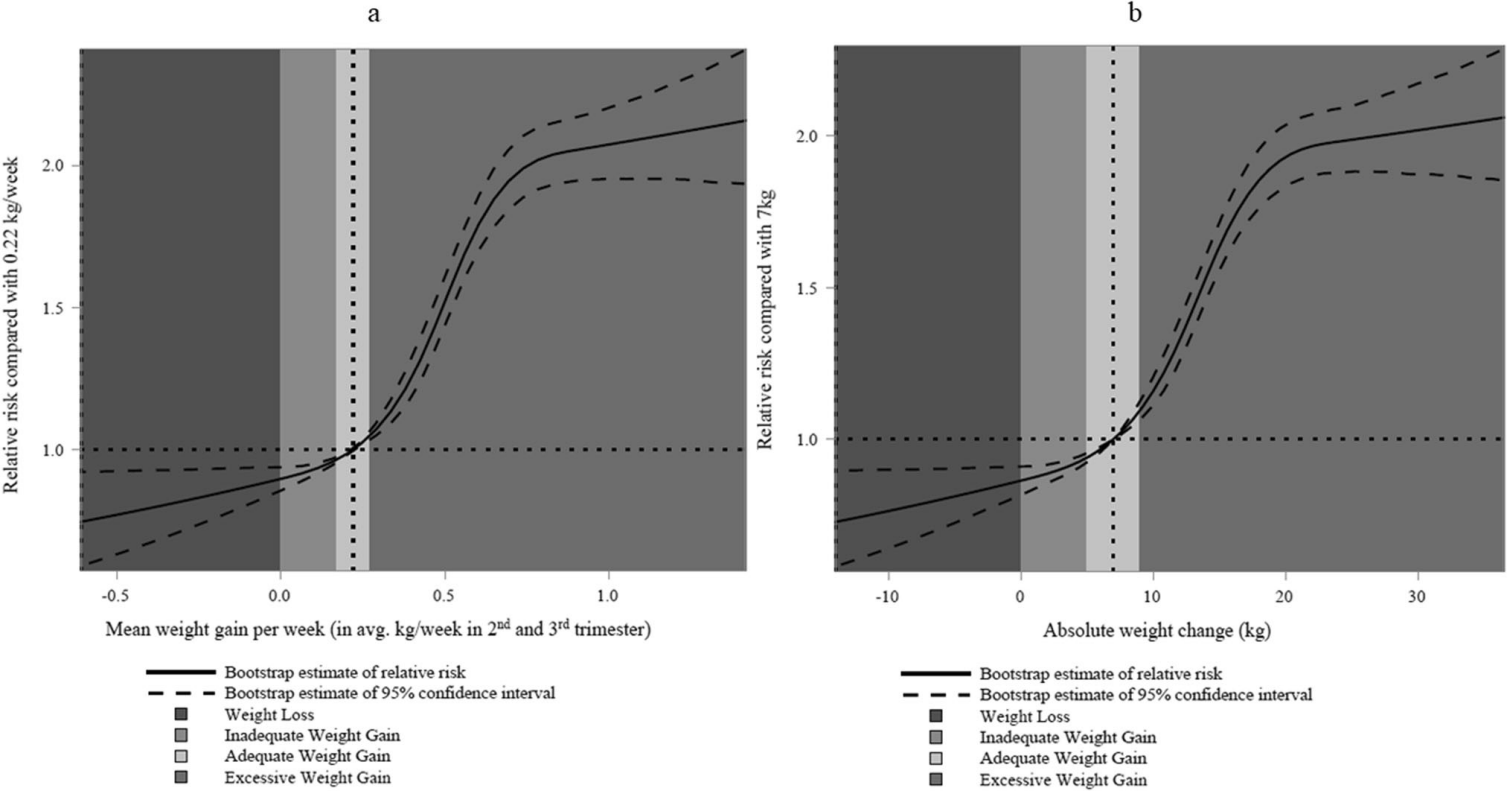
Dose-response relationship between weight change and risk of LGA (>90th percentile) neonates. **a**, **b**. Dose-response relationship between weight change during pregnancy and risk of LGA (>90th percentile) neonates. RRs and 95% CIs of LGA (>90th percentile) were calculated with respect to total gestational weight change in kilograms (**a**) and average gestational weight change per week (**b**). Results from multivariable regression models using generalized estimation equation (GEE) methods and restricted cubic splines with 5 knots. Model adjusted for maternal age, gestational age, pre-pregnancy BMI, parity, neighbourhood household median income quintile, smoking, and antenatal health care provider. Each plot was centred to display 99% of gestational weight change values in the graph.

Regarding the non-linear relationship between gestational weight change and risk of SGA and LGA neonates, restricted cubic spline regression analysis revealed that total gestational weight change displayed non-linear L-shaped associations with SGA (<10th percentile) neonates, showing an increased risk of SGA (<10th percentile) with increased GWL (Fig. 4a, b). Conversely, gestational weight change displayed non-linear S-shaped associations with LGA (>90th percentile) neonates, with a decreased risk of LGA (>90th percentile) observed along with increased GWL (Fig. 5a, b). Supplementary Figures 1 and 2 show the overall results for SGA <3rd percentile and LGA >97th percentile, respectively. Similar shapes were observed in obesity classes I and II. For the obesity class III+, a non-linear U shape was observed for SGA (<10th percentile), and an inversed U shape was found for LGA (>90th percentile) (Supplementary Figs. 3–8). Examining GWL alone, we found that increased GWL was associated with a tendency towards a higher risk of SGA neonates and a lower risk of LGA neonates, regardless of obesity class.

## Discussion

The findings of this large population-based study showed that, in women with obesity who had uncomplicated pregnancies, GWL was generally associated with an increased risk of SGA neonates (aRR: 1.45, 95% CI: 1.30–1.60) and decreased risk of LGA neonates (aRR: 0.81, 95% CI: 0.69–0.94), compared to those with adequate gestational weight gain. Additionally, the non-linear restricted cubic spline curves demonstrated an L-shaped curve for SGA neonates and an S-shaped curve for LGA neonates, revealing an increased risk of SGA neonates and a decreased risk of LGA with higher GWL. These findings support the previous observations [26] and contribute to a deeper understanding of the dose-response relationship between GWL and fetal growth.

The precise mechanism behind GWL resulting in abnormal fetal growth is not fully understood. During pregnancy, women are expected to increase their energy intake (i.e., diet) to support the process of fetal and maternal tissue growth, irrespective of their pre-pregnancy BMI, [27]. This increase in energy requirements is mainly due to weight gain and higher metabolic rate associated with maternal cardiac output and fetal metabolism throughout mid and late pregnancy [28]. However, a study that evaluated energy balance during pregnancy found that the mobilization of maternal fat mass in pregnant women with obesity compensates for the energy demand produced by the pregnancy and growing fetus [28, 29]. The magnitude of energy imbalance is an important factor in achieving adequate gestational weight gain, and preventing pregnancy-related complications such as SGA and LGA neonates [29]. Although the rates of neonates with SGA have decreased internationally and in Canada (from 11.1% to 7.2% between 1978 and 1996) [21], divergent fetal growth is still associated with severe outcomes, including hypoxic and traumatic composites of neonate morbidity [30].

SGA is an important indicator of fetal morbidity and mortality. SGA neonates can face significant fetal growth complications, including long-term effects on child development such as an increased risk of cardiovascular disease, high blood pressure, and impacts on cognition and intellectual ability [31–33]. Our study findings showed that women with obesity, regardless of the obesity class (I, II, or III+), have a greater risk of having SGA neonates when they experience GWL. While very limited studies have examined the impact of GWL among women with obesity prior to pregnancy. A systematic review reported that women with GWL had higher odds of SGA neonates (<10th percentile) (adjusted odds ratio [aOR]: 1.76, 95% CI: 1.45–2.14) [11]. In contrast to our study, a trend towards a graded relationship between SGA <10th percentile and each of the three obesity groups (I: aOR: 1.73, 95% CI: 1.53–1.97; II: aOR: 1.63, 95% CI: 1.44–1.85; and III: aOR: 1.39, 95% CI, 1.17–1.66, respectively) was observed in this previous study. This suggests that women with a higher BMI (e.g., obesity class III) had a decreased risk of having SGA neonates when compared with those with a lower BMI (e.g., obesity class I). A more recent study that looked at GWL among women with obesity class II found an increased incidence of SGA <10th percentile but not of <3rd percentile, compared to adequate gestational weight gain [34]. However, the primary limitation of these studies is that they were not able to exclude the medical conditions that could lead to unintended GWL. Additionally, most of these studies sample size was not powered to produce expected results.

In terms of LGA neonates, the association between GWL or inadequate gestational weight gain among women with obesity and lower rates of LGA neonates has been reported. LGA neonates are at risk of several complications, including hypoglycemia, birth trauma, and long-term obesity [35–37]. However, the association between GWL and LGA neonates in the population living with obesity needs further exploration. Although the systematic review by Kapadia et al. reported lower odds of LGA neonates (>90th percentile) (aOR: 0.57, 95% CI: 0.52–0.62) in women with GWL [11], data from the Swedish Medical Birth Registry suggested that it may be reasonably safe for women with obese BMI (class II and III) to lose weight during pregnancy [9]. Blomberg et al. found that the risk of LGA neonates decreases or remains unaffected among women with GWL [9]. However, previous studies did not fully consider some relevant confounders in their analysis. After adjusting for important confounders and removing cases with medical conditions leading to weight loss, our regression analysis suggests that GWL is associated with a decreased risk of LGA neonates in women with overall obesity, specifically in those with class II or higher obesity. The non-linear cubic spline curves confirm the trend shown in the regression analysis. However, results for women with obesity class III+ must be interpreted with caution due to the small sample size and increased risk of placental dysfunction in this population that could affect birthweight.

This study has several strengths. First, we identified and excluded several medical conditions that could cause unintentional weight loss during pregnancy. Excluding women with those medical conditions from the analysis helped to reduce the confounding bias. We also took important confounders into consideration in the analysis, including the maternal age, gestational age, pre-pregnancy BMI, parity, neighbourhood household median income quintile, smoking, and antenatal health care provider. Furthermore, we used multivariable regression models with restricted cubic splines to precisely estimate the association between GWL and the risk of SGA and LGA neonates among women with obesity overall and per BMI group. To the best of our knowledge, this is the first cohort study to investigate GWL among women with high BMI, analyzing over 50,000 women.

However, the main limitation of this study is the lack of valid and accurate measurements of maternal weight and height in administrative databases. The registry collects self-reported data on pre-pregnancy weight and weight at delivery, which may lead to underestimation or overestimation of the prevalence of obesity and gestational weight gain or loss. Although, research has shown that women with higher BMI are more likely to underestimate their self-reported weight [38, 39], highly correlated data have been found between self-reported and measured values in BMI and gestational weight change [40–42]. Although using self-reported pre-pregnancy obesity may slightly underestimate our outcome, the general trend of association between pregnancy weight and perinatal outcomes may be unaffected [38]. Other limitations of the present study include the lack of data on ethnicity and history of SGA and LGA. Nonetheless, the lack of ethnicity and history of SGA and LGA data in the database adds bias to our results, as these are relevant confounders of the studied association.

In conclusion, our study provides evidence of an increased risk of SGA neonates and a reduced risk of LGA neonates among women with obesity who experience GWL. However, due to the risks associated with SGA neonates, recommendations around GWL for women with obesity should be interpreted with caution.

### Supplementary information

Supplemental Material

## Data Availability

The data analyzed during this study are held securely at the prescribed registry BORN Ontario. Data sharing regulations prevent these data from being made available publicly due to the personal health information in the datasets. Enquiries regarding BORN data must be directed to BORN Ontario (Science@BORNOntario.ca).
