# Supplementary material for: Gestational weight loss and fetal growth in uncomplicated pregnancies among women with obesity: a population-based retrospective cohort study
Source: Int J Obes (Lond). 2023 Oct 13;47(12):1269–77. doi: 10.1038/s41366-023-01382-6 (PMC10663149; doi:10.1038/s41366-023-01382-6)
Supplement: Supplementary file 1 — Supplemental Material [file 41366_2023_1382_MOESM1_ESM.docx]

**Supplementary Material**

**Tables**

**Supplementary Table 1.** The maternal conditions that could cause unintended significant weight loss during pregnancy

| **Maternal conditions** | **BORN codes** | **DAD (ICD-10-CA) codes** |
| --- | --- | --- |
| Tuberculosis |  | A15. 7 &16.7 |
| Hepatitis |  | B15-B19 |
| HIV |  | B20 |
| Pre-exiting hypertension | N0016 |  |
| Pre-existing diabetes | D0013 |  |
| Autoimmune disorders | M0013 |  |
| Hyperthyroidism | M0013 |  |
| Hypothyroidism | M0013 |  |
| Cancers | M0013 |  |
| Endocrine imbalance | M0013 |  |
| Infections | D0017 |  |
| Anxiety | M0048 |  |
| Depression | M0048 |  |
| Eating disorders |  | F50.9 |
| Gastrointestinal diseases |  | K92.9 |

**Supplementary Table 2.** Association between weight change during pregnancy and risk of SGA (<10^th^ percentile) and LGA (>90^th^ percentile) neonates by obesity class

|  | **Complete case analysis** | | | | | |
| --- | --- | --- | --- | --- | --- | --- |
|  | **SGA<10^th^ percentile** | | | **LGA>90^th^ percentile** | | |
|  | **n** | **%** | **adjusted RR** | **n** | **%** | **adjusted RR** |
| **Overall ^a^** |  |  |  |  |  |  |
| Weight loss | 274 | 10.0 | 1.45 (1.24, 1.69) | 316 | 11.5 | 0.80 (0.70, 0.91) |
| Inadequate weight gain | 502 | 8.4 | 1.21 (1.06, 1.38) | 690 | 11.5 | 0.81 (0.73, 0.89) |
| Adequate weight gain | 421 | 6.9 | Ref | 833 | 13.6 | Ref |
| Excessive weight gain | 1,908 | 5.1 | 0.70 (0.62, 0.78) | 6,869 | 18.5 | 1.44 (1.34, 1.55) |
| Total | 3,105 | 6.0 |  | 8,708 | 16.8 |  |
| **Class I obesity ^b^** |  |  |  |  |  |  |
| Weight loss | 143 | 12.2 | 1.52 (1.24, 1.86) | 108 | 9.2 | 0.93 (0.75, 1.15) |
| Inadequate weight gain | 296 | 9.8 | 1.25 (1.06, 1.48) | 269 | 8.9 | 0.83 (0.71, 0.98) |
| Adequate weight gain | 278 | 7.8 | Ref | 379 | 10.6 | Ref |
| Excessive weight gain | 1,328 | 5.3 | 0.66 (0.57, 0.76) | 4,188 | 16.8 | 1.64 (1.47, 1.82) |
| **Class II obesity ^b^** |  |  |  |  |  |  |
| Weight loss | 69 | 8.5 | 1.40 (1.03, 1.90) | 99 | 12.1 | 0.74 (0.59, 0.93) |
| Inadequate weight gain | 129 | 7.3 | 1.16 (0.89, 1.52) | 238 | 13.5 | 0.81 (0.68, 0.96) |
| Adequate weight gain | 102 | 6.0 | Ref | 278 | 16.4 | Ref |
| Excessive weight gain | 378 | 4.6 | 0.73 (0.58, 0.91) | 1,757 | 21.4 | 1.35 (1.19, 1.52) |
| **Class III or greater obesity ^b^** |  |  |  |  |  |  |
| Weight loss | 62 | 8.1 | 1.55 (1.05, 2.30) | 109 | 14.2 | 0.65 (0.51, 0.81) |
| Inadequate weight gain | 77 | 6.4 | 1.24 (0.84, 1.81) | 183 | 15.1 | 0.69 (0.57, 0.84) |
| Adequate weight gain | 41 | 4.8 | Ref | 176 | 20.6 | Ref |
| Excessive weight gain | 202 | 5.1 | 0.97 (0.69, 1.36) | 924 | 23.5 | 1.14 (0.98, 1.32) |

Data source: BORN-CIHI linked data 2012-2017

^a^ Models were adjusted for maternal age, gestational age, pre-pregnancy BMI, parity, neighbourhood-level income, smoking, and antenatal health care provider.

^b^ Models were adjusted for maternal age, gestational age, parity, neighbourhood-level income, smoking, and antenatal health care provider.

Multiple imputations methods were used to account for the missing data of covariates and confounders in the regression models. Five complete datasets were imputed using the fully conditional specification method (FCS) method. Maternal age, gestational age, and pre-pregnancy BMI were imputed using a linear regression model. Antenatal healthcare providers, parity, neighbourhood-level income, and smoking were imputed using logistic regression models.

**Supplementary Table 3**. Association between weight change during pregnancy and risk of SGA (<3^rd^ percentile) and LGA (>97^th^ percentile) neonates

|  | **Complete case analysis** | | | | | | **Imputed data results** | | |  |
| --- | --- | --- | --- | --- | --- | --- | --- | --- | --- | --- |
|  | **SGA<3^rd^**  **percentile** | | | **LGA>97^th^**  **percentile** | | | **SGA <3^rd^ percentile** | **LGA >97^th^ percentile** | |  |
|  | **n** | **%** | **adjusted RR** | **n** | **%** | **adjusted**  **RR** | **adjusted RR** | | **adjusted RR** | |
| Weight loss | 83 | 3.0 | 1.54  (1.15, 2.08) | 97 | 3.5 | 0.79  (0.62, 1.01) | 1.59  (1.30, 1.88) | | 0.82  (0.59, 1.06) | |
| Inadequate weight gain | 153 | 2.6 | 1.34  (1.04, 1.73) | 230 | 3.8 | 0.89  (0.73, 1.07) | 1.38  (1.13, 1.62) | | 0.93  (0.75, 1.11) | |
| Adequate weight gain | 114 | 1.9 | Ref | 244 | 4.0 | Ref | Ref | | Ref | |
| Excessive weight gain | 465 | 1.3 | 0.62  (0.50, 0.78) | 2,507 | 6.8 | 1.83  (1.60, 2.09) | 0.61  (0.40, 0.82) | | 1.86  (1.73, 2.00) | |
| Total | 815 | 1.6 |  | 3,078 | 5.9 |  |  | |  | |

Data source: BORN-CIHI linked data 2012-2017

Uncomplicated pregnancy is defined as a pregnant woman with singleton birth without any following conditions: lethal anomalies, tuberculosis, hepatitis, HIV, cancer, hypertension, autoimmune disorder, diabetes, hypothyroidism, hyperthyroidism or other endocrine disorder, anxiety, and depression.

Models were adjusted for maternal age, gestational age, pre-pregnancy BMI, parity, neighbourhood-level income, smoking, and antenatal health care provider.

Multiple imputations methods were used to account for the missing data of covariates and confounders in the regression models. Five complete datasets were imputed using the fully conditional specification method (FCS) method. Maternal age, gestational age, and pre-pregnancy BMI were imputed using a linear regression model, Antenatal healthcare provider, parity, neighbourhood-level income, and smoking were imputed using logistic regression models.

**Supplementary Table 4.** Rates of SGA <3^rd^ percentile and LGA >97^th^ percentile by weight change during pregnancy and obesity class

|  | SGA <3^rd^ percentile | | LGA >97^th^ percentile | |
| --- | --- | --- | --- | --- |
|  | **n** | **%** | **n** | **%** |
| **Overall** |  |  |  |  |
| Weight loss | 83 | 3.0 | 97 | 3.5 |
| Inadequate weight gain | 153 | 2.6 | 230 | 3.8 |
| Adequate weight gain | 114 | 1.9 | 244 | 4.0 |
| Excessive weight gain | 465 | 1.3 | 2,507 | 6.8 |
| Total | 815 | 1.6 | 3,078 | 5.9 |
| **Class I obesity** |  |  |  |  |
| Weight loss | 46 | 3.9 | 33 | 2.8 |
| Inadequate weight gain | 85 | 2.8 | 85 | 2.8 |
| Adequate weight gain | 81 | 2.3 | 107 | 3.0 |
| Excessive weight gain | 328 | 1.3 | 1,415 | 5.7 |
| Total | 540 | 1.7 | 1,640 | 5.0 |
| **Class II obesity** |  |  |  |  |
| Weight loss | 18 | 2.2 | 26 | 3.2 |
| Inadequate weight gain | 41 | 2.3 | 82 | 4.7 |
| Adequate weight gain | 20 | 1.2 | 88 | 5.2 |
| Excessive weight gain | 87 | 1.1 | 683 | 8.3 |
| Total | 166 | 1.3 | 879 | 7.0 |
| **Class III or greater obesity** |  |  |  |  |
| Weight loss | 19 | 2.5 | 38 | 5.0 |
| Inadequate weight gain | 27 | 2.2 | 63 | 5.2 |
| Adequate weight gain | 13 | 1.5 | 49 | 5.7 |
| Excessive weight gain | 50 | 1.3 | 409 | 10.4 |
| Total | 109 | 1.6 | 559 | 8.3 |

Data source: BORN-CIHI linked data 2012-2017

**Figures**

**Supplementary Figure 1.** Dose-response relationship between weight change during pregnancy and risk of SGA (<3^rd^ percentile) neonates


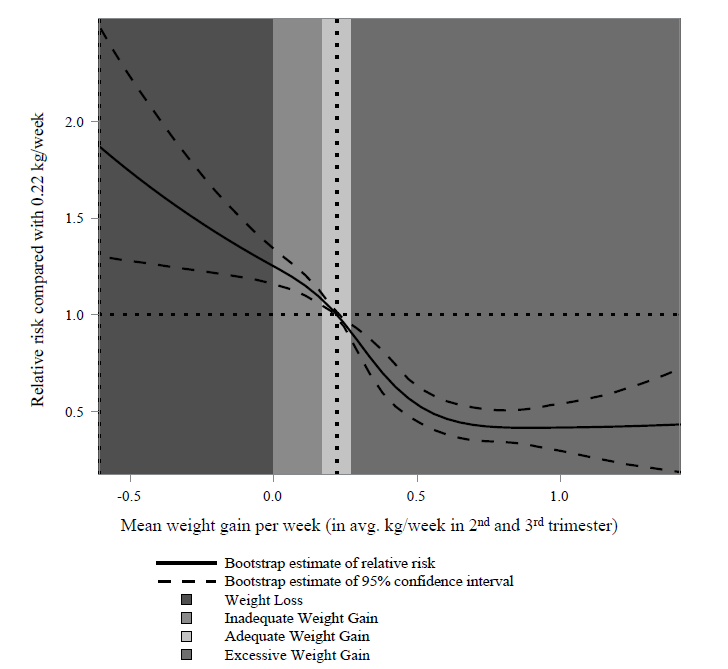


RRs and 95% CIs of SGA (<3rd percentile) were calculated with respect to total gestational weight change in kilograms (a) and average gestational weight change per week (b). Results from multivariable regression models using GEEs and restricted cubic splines with 5 knots. Model adjusted for maternal age, gestational age, pre-pregnancy BMI, parity, neighbourhood household median income quintile, smoking, and antenatal health care provider. Each plot was centred to display 99% of gestational weight change values in the graph.

**Supplementary Figure 2.** Dose-response relationship between weight change during pregnancy and risk of LGA (>97^th^ percentile) neonates


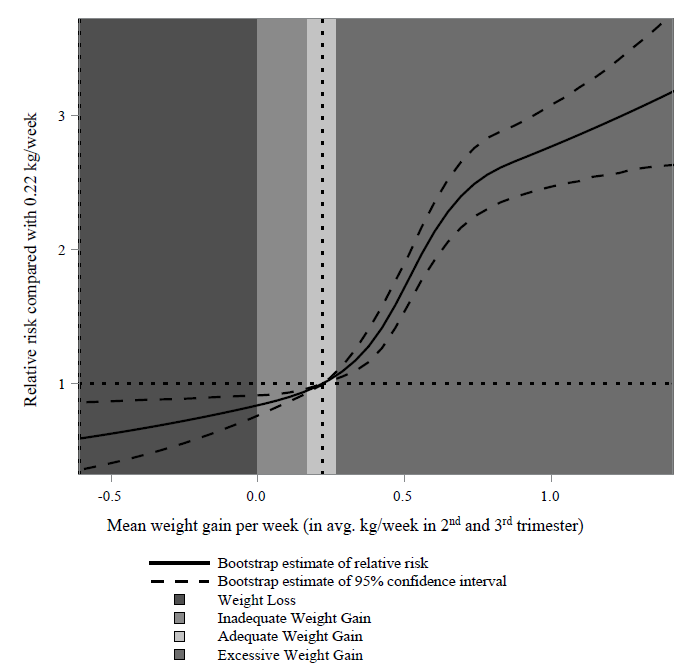


RRs and 95% CIs of LGA (>97th percentile) were calculated with respect to total gestational weight change in kilograms (a) and average gestational weight change per week (b). Results from multivariable regression models using GEEs and restricted cubic splines with 5 knots. Model adjusted for maternal age, gestational age, pre-pregnancy BMI, parity, neighbourhood household median income quintile, smoking, and antenatal health care provider. Each plot was centred to display 99% of gestational weight change values in the graph.

**Supplementary Figure 3.** Dose-response relationship between weight change during pregnancy and risk of SGA (<10^th^ percentile) neonates for women with obesity class I


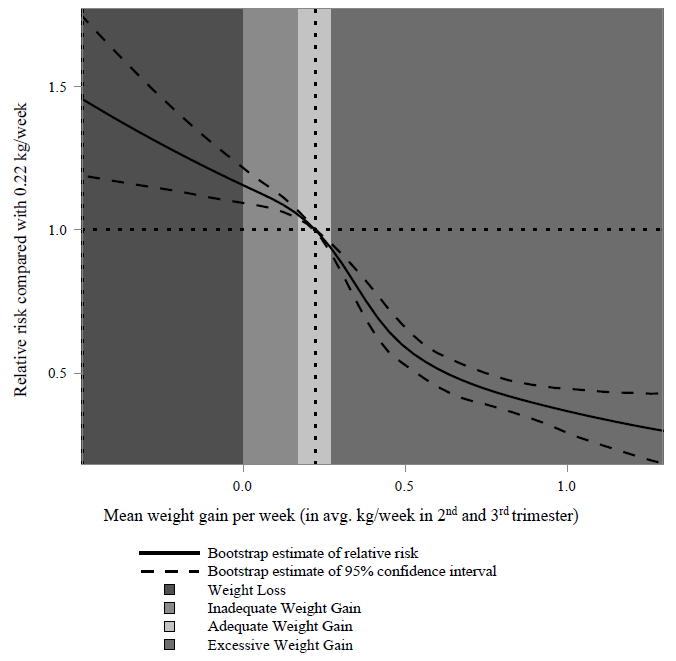


RRs and 95% CIs of SGA (<10th percentile) were calculated with respect to total gestational weight change in kilograms (a) and average gestational weight change per week (b). Results from multivariable regression models using GEEs and restricted cubic splines with 5 knots. Model adjusted for maternal age, gestational age, pre-pregnancy BMI, parity, neighbourhood household median income quintile, smoking, and antenatal health care provider. Each plot was centred to display 99% of gestational weight change values in the graph.

**Supplementary Figure 4.** Dose-response relationship between weight change during pregnancy and risk of SGA (<10^th^ percentile) neonates for women with obesity class II


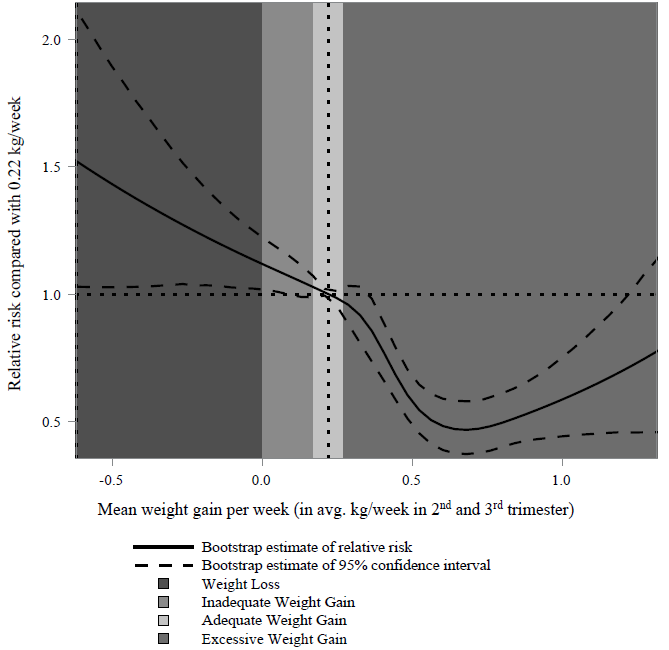


RRs and 95% CIs of SGA (<10th percentile) were calculated with respect to total gestational weight change in kilograms (a) and average gestational weight change per week (b). Results from multivariable regression models using GEEs and restricted cubic splines with 5 knots. Model adjusted for maternal age, gestational age, pre-pregnancy BMI, parity, neighbourhood household median income quintile, smoking, and antenatal health care provider. Each plot was centred to display 99% of gestational weight change values in the graph.

**Supplementary Figure 5.** Dose-response relationship between weight change during pregnancy and risk of SGA (<10^th^ percentile) neonates for women with obesity class III+


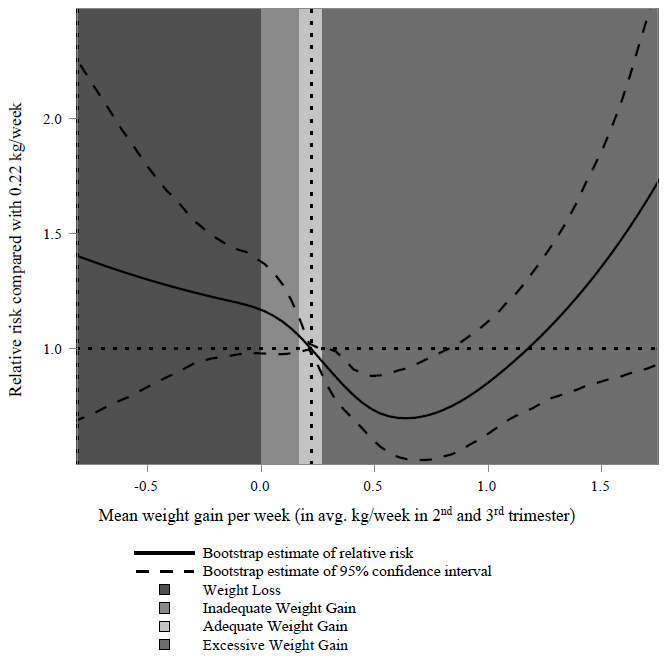


RRs and 95% CIs of SGA (<10th percentile) were calculated with respect to total gestational weight change in kilograms (a) and average gestational weight change per week (b). Results from multivariable regression models using GEEs and restricted cubic splines with 5 knots. Model adjusted for maternal age, gestational age, pre-pregnancy BMI, parity, neighbourhood household median income quintile, smoking, and antenatal health care provider. Each plot was centred to display 99% of gestational weight change values in the graph.

**Supplementary Figure 6.** Dose-response relationship between weight change during pregnancy and risk of LGA (>90^th^ percentile) neonates for women with obesity class I


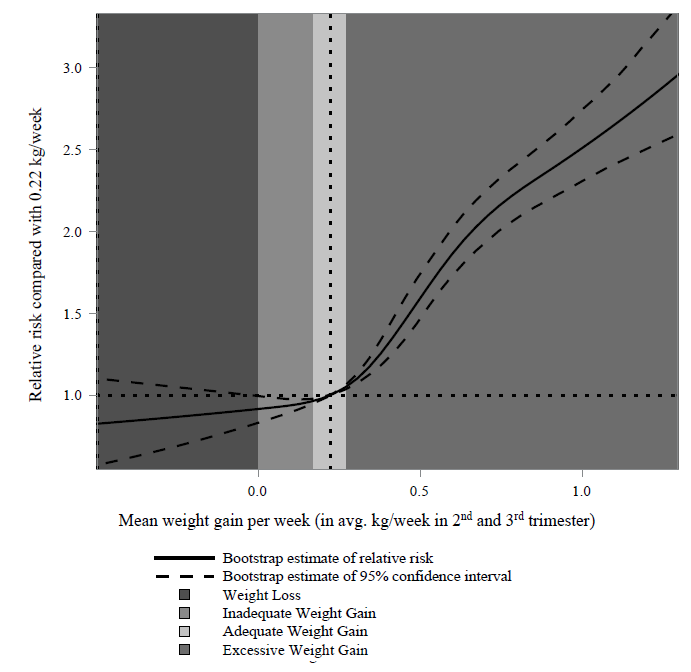


RRs and 95% CIs of LGA (>90th percentile) were calculated with respect to total gestational weight change in kilograms (a) and average gestational weight change per week (b). Results from multivariable regression models using GEEs and restricted cubic splines with 5 knots. Model adjusted for maternal age, gestational age, pre-pregnancy BMI, parity, neighbourhood household median income quintile, smoking, and antenatal health care provider. Each plot was centred to display 99% of gestational weight change values in the graph.

**Supplementary Figure 7.** Dose-response relationship between weight change during pregnancy and risk of LGA (>90^th^ percentile) neonates for women with obesity class II


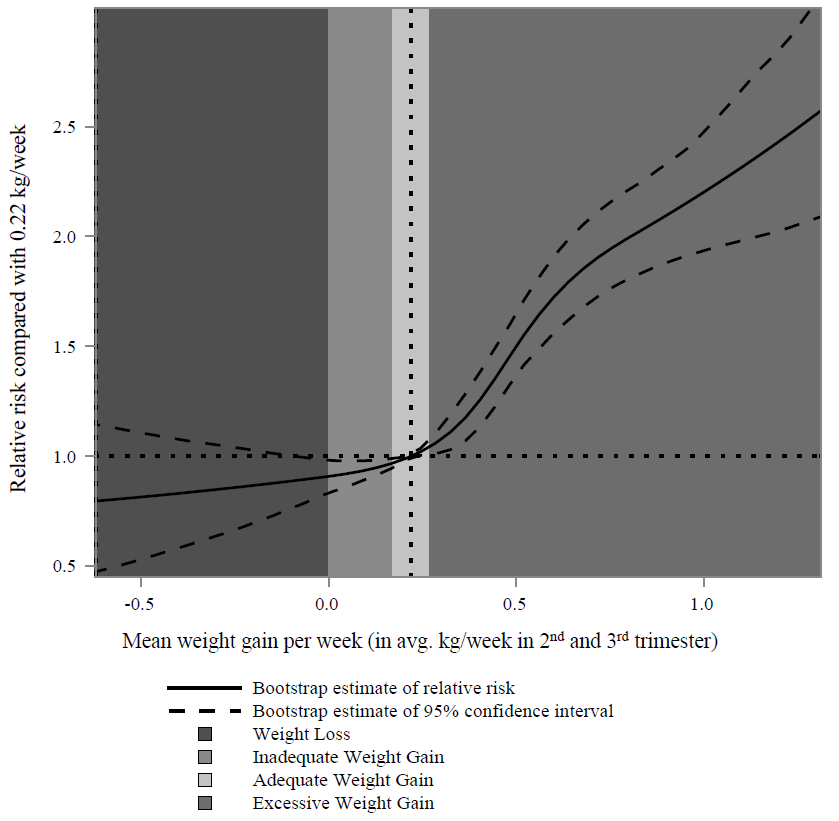


RRs and 95% CIs of LGA (>90th percentile) were calculated with respect to total gestational weight change in kilograms (a) and average gestational weight change per week (b). Results from multivariable regression models using GEEs and restricted cubic splines with 5 knots. Model adjusted for maternal age, gestational age, pre-pregnancy BMI, parity, neighbourhood household median income quintile, smoking, and antenatal health care provider. Each plot was centred to display 99% of gestational weight change values in the graph.

**Supplementary Figure 8.** Dose-response relationship between weight change during pregnancy and risk of LGA (>90^th^ percentile) neonates for women with obesity class III+

**
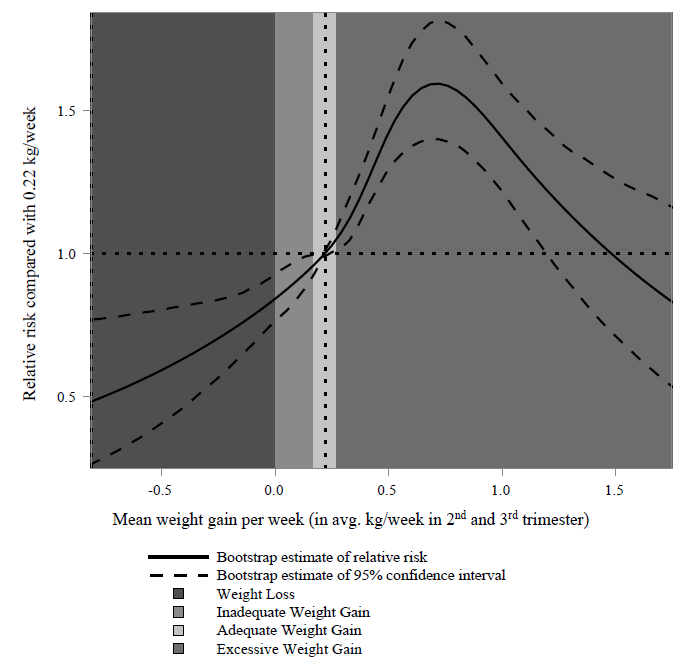
**

RRs and 95% CIs of LGA (>90th percentile) were calculated with respect to total gestational weight change in kilograms (a) and average gestational weight change per week (b). Results from multivariable regression models using GEEs and restricted cubic splines with 5 knots. Model adjusted for maternal age, gestational age, pre-pregnancy BMI, parity, neighbourhood household median income quintile, smoking, and antenatal health care provider. Each plot was centred to display 99% of gestational weight change values in the graph.
